# When Do Financial Incentives Reduce Intrinsic Motivation? Comparing Behaviors Studied in Psychological and Economic Literatures

**DOI:** 10.1037/a0032727

**Published:** 2013-09

**Authors:** Marianne Promberger, Theresa M. Marteau

**Affiliations:** 1 Centre for the Study of Incentives in Health and Department of Psychology, King’s College London, London, United Kingdom

**Keywords:** incentives, health behavior, motivation, motivation crowding out, review

## Abstract

***Objective:*** To review existing evidence on the potential of incentives to undermine or “crowd out” intrinsic motivation, in order to establish whether and when it predicts financial incentives to crowd out motivation for health-related behaviors. ***Method:*** We conducted a conceptual analysis to compare definitions and operationalizations of the effect, and reviewed existing evidence to identify potential moderators of the effect. ***Results:*** In the psychological literature, we find strong evidence for an undermining effect of tangible rewards on intrinsic motivation for simple tasks when motivation manifest in behavior is initially high. In the economic literature, evidence for undermining effects exists for a broader variety of behaviors, in settings that involve a conflict of interest between parties. By contrast, for health related behaviors, baseline levels of incentivized behaviors are usually low, and only a subset involve an interpersonal conflict of interest. Correspondingly, we find no evidence for crowding out of incentivized health behaviors. ***Conclusion:*** The existing evidence does not warrant a priori predictions that an undermining effect would be found for health-related behaviors. Health-related behaviors and incentives schemes differ greatly in moderating characteristics, which should be the focus of future research.

Policymakers are increasingly interested in the potential of financial incentives to change individual behavior to achieve health-related outcomes, including smoking cessation, weight loss, attendance for disease screening, undergoing vaccination, or donating blood. Such incentive schemes aimed at individuals or patients are frequently met with criticism by the general public and professional groups (e.g., Parke, Ashcroft, Brown, Marteau, & Seale, 2011; Promberger, Brown, Ashcroft, & Marteau, 2011). One concern about using financial incentives to change health-related behaviors is that they may undermine, or “crowd out,” intrinsic motivation for the behavior. For example, paying someone to lose weight might make that person less motivated to lose weight once the incentive is removed. This concern is usually raised with reference to one or more of three distinct, but related, literatures: Titmuss’s (1970) *The Gift Relationship*, claiming detrimental effects of paying for blood donation, psychological literature starting with Deci (1971) on the undermining effect of rewards on intrinsic motivation, and economic literature about motivation crowding out or “motivation crowding theory” (Frey, 1997; Frey & Jegen, 2001). Although there is little empirical evidence for Titmuss’s claim, the psychological and economic literatures comprise a large body of laboratory and field studies attempting to demonstrate unintended consequences of incentives.

It is sometimes assumed that the evidence base from these literatures applies directly to the use of incentives to change health related behaviors, implying that external rewards likely undermine motivation for these behaviors. For example, Johnston and Sniehotta (2010) state, with reference to Deci, Koestner, and Ryan (1999) “[t]here is good evidence from Self-Determination Theory that invoking ‘intrinsic motivation’ (i.e., based on the patient’s own values) results in more sustainable behavior change after the intervention ends, than those which only arouse ‘external motivation’” (p. 131).

The aim of the present article is twofold. First, we aim to provide an overview of the literature on incentives for health-related behaviors to establish whether an undermining effect of rewards has been found to occur for these behaviors.

Second, we aim to compare the findings from this overview with the existing evidence from the psychological literature on rewards undermining intrinsic motivation and the economic literature around motivation crowding out. We compare the evidence from these fields to characterize the behaviors and contexts in which an undermining effect has been observed, and where it has not (see Table 1).[Table-anchor tbl1]

## What Are Health-Related Behaviors?

What do we mean by health-related behaviors? For this article, we are interested in behaviors for which evidence exists that they impact on health-related outcomes, and which for this reason are the focus of public policy intervention to change individuals’ behavior. These include smoking cessation, weight loss, physical activity, medication adherence, vaccination, screening for treatable or preventable disease, and blood and organ donation. These behaviors obviously differ on a range of characteristics, including how they affect health outcomes, who benefits from the behavior (the individual or others), whether health benefits are realized sooner or later, whether the behavior needs to be sustained to accrue benefits, and in how any financial incentive system would plausibly be implemented, for example, whether it would be tied to the behavior such as attending for screening, or a specified outcome, such as weight loss.

Before we focus on the evidence regarding these behaviors, we give an overview over the existing psychological and economic literatures on the potentially undermining effect of rewards; see Table 1 for an overview.

## Psychological Literature on Rewards Undermining Intrinsic Motivation

By 1970 the reinforcing effect of rewards on behavior was firmly established in the learning theory literature. Deci (1971) and Lepper, Greene, and Nisbett (1973) investigated a possible exception to this effect: whether rewards could have a detrimental effect if they were offered for an activity that the individual enjoyed. The hypothesis was that rewards might undermine “intrinsic motivation,” tested by comparing behavior levels between a group that received a tangible reward and a group that received no such reward, after the reward had been removed. Both studies found that behavior levels were lower in the group that had previously been rewarded. “Intrinsic motivation” was defined as performance of a task with “no apparent rewards except the activity itself” (Deci, 1971, p. 105) and operationalized by choosing tasks that study participants already performed with sufficiently high persistence (puzzle-solving and writing student newspaper headlines), and by selecting as participants about 50% of nursery schoolchildren who showed the highest initial spontaneous interest in a new drawing opportunity (Lepper et al., 1973).

A wide body of research examined the prevalence of the undermining effect of rewards, with meta-analyses including 124 studies (Cameron & Pierce, 1994) and 128 studies (Deci et al., 1999). Virtually all these studies examine the effect of rewards on subjects’ persistence in simple tasks such as puzzles or drawing pictures, with participants being children (mostly preschool or elementary school) in about half the studies, college students in the others (Deci et al., 1999, Tables 1–6).

These meta-analyses come to different conclusions, either that the undermining effect of tangible external rewards on intrinsic motivation is pervasive (Deci et al., 1999; Deci, Koestner, & Ryan, 2001), or that it is limited (Cameron, 2001; Cameron, Banko, & Pierce, 2001; Cameron & Pierce, 1994). This difference seems to largely depend on methodological decisions, in particular which studies to include in the meta-analyses, and which study characteristics are considered to be moderators. Of particular relevance to the question of crowding out in health contexts is that the rewards have no undermining or even a positive effect on “dull,” “boring,” “uninteresting” tasks, that is, tasks for which initial intrinsic motivation is low, operationalized by low initial task activity. In countering Cameron’s (2001) argument for their inclusion, Deci, Ryan, and Koestner (2001) specifically point out that the research field of external rewards on intrinsic motivation “has always been defined in terms of reward effects on intrinsic motivation for interesting activities,” an argument supported by authors not directly involved in the debate (Lepper, Henderlong, & Gingras, 1999). Following the original definition and operationalization of intrinsic motivation, high levels of pre-reward behavior are assumed to imply high intrinsic motivation.

Cognitive evaluation theory (CET; Deci & Ryan, 1985) predicts that rewards only reduce intrinsic motivation when they are perceived as controlling the behavior, and might actually enhance intrinsic motivation if they engender feelings of competence. In line with this prediction, no reduction of intrinsic motivation is found if the reward is not tangible, for example, if it comprises verbal praise, and if it is unexpected (Cameron & Pierce, 1994; Deci et al., 1999).

## Economic Literature on “Motivation Crowding Out”

The term “motivation crowding out” was coined in the economic literature to refer to an undermining effect of rewards and its definition extended to any effect that is opposite to the relative price effect of standard economic theory, whereby reduced costs should increase behavior, and increased costs should reduce it. The effect, therefore, includes cases where penalties increase behavior, and focuses on behavior concurrent with the incentive rather than after its removal.

Economic studies on motivation crowding out include new and often more complex behaviors than the puzzles and tasks used in the cognitive evaluation theory literature. Laboratory studies often use economic games that require some cooperation between parties or a trade-off between interests of different parties, for example in principal-agent problems involving an agent acting with consequences for the principal, who relies on the agent, such as an employer relying on employee work effort. Questionnaire measures of real-world voting decisions have also been used: In a within-subject design, Frey and Oberholzer-Ghee (1997) found acceptance of the siting of a nuclear waste repository close by dropped from about 50% to about 25% when financial compensation was offered. Field experiments showed, for example, parents to be more often late in picking up their children from daycare when a fine was imposed than before (and even after the fine was removed; Gneezy & Rustichini, 2000), and volunteers who were paid a small amount worked fewer hours than unpaid volunteers (Frey & Goette, 1999).

Frey and Jegen’s (2001) motivation crowding theory builds on cognitive evaluation theory and also includes the perception of the incentive as controlling or undermining autonomy as a mechanism that leads to motivation crowing out. It also explicitly allows for the possibility of extrinsic incentives to “crowd in,” or increase, intrinsic motivation, for example through the development of new preferences favoring the incentivized behavior.

Economic research currently lags behind the psychological literature as measured by the sheer volume of studies. Meta-analyses are not available. The most comprehensive unsystematic review to date (Frey & Jegen, 2001), including empirical studies as well as anecdotal evidence, concludes that rewards may sometimes crowd out and sometimes crowd in—that is, increase—intrinsic motivation. Economic research about crowding out is conducted given the strong theoretical and empirically well-founded backdrop of the relative price effect: Ample evidence exists that behavior is usually in line with incentives (e.g., Prendergast, 1999), and research on motivation crowding out is looking for the exceptions. Although increasing numbers of studies uncover such exceptions, the conditions under which they occur are currently not well understood (Gneezy, Meier, & Rey-Biel, 2011).

## Results

### Evidence From Studies About Health-Related Behaviors

#### Definition and operationalization of an undermining effect of rewards on motivation

In interpreting the evidence for an undermining effect of rewards on health-related behaviors, we will rely both on the definition and operationalization used in the psychological cognitive evaluation theory and in the economic motivation crowding out literature. Both literatures focus on a difference in levels of behavior between the incentivized group and a non-incentivized control group when operationalizing an undermining effect of rewards and a motivation crowding out effect, respectively. The CET literature focuses on the difference after removal of the reward: After an initial period of pre-reward task activity (sometimes omitted), the experimental group is rewarded for the task; subsequently rewards are removed, and time spent on the task is compared between the experimental and a control group in a “free-choice” period. Less time spent on the task in the previously rewarded group during this period than in the control group indicates that the reward has reduced intrinsic motivation.

The definition of “motivation crowding out” in economic research includes any effect that is opposite to the relative price effect of standard economic theory: rewards decreasing behavior levels, and penalties increasing behavior levels. Correspondingly, the economic literature includes operationalizations of a crowding out effect as the difference in behavior between an incentivized and a control group while the incentive is in place (Frey, 1997; Frey & Jegen, 2001).

The focus in both literatures on differences in behavior as the relevant outcome means we can look for evidence of an undermining effect of rewards even in health-related behavior studies which do not set out to look for this effect: In randomized controlled trials of health behavior incentives, evidence for the effectiveness of the incentive provides evidence for the absence of motivation crowding out as defined and operationalized by economists. If the incentivized and the control groups are compared during follow-up—that is, after the removal of incentives,—evidence for motivation crowding out would require lower levels of the previously incentivized behavior in the previously incentivized group than in the control group.

#### Studies explicitly addressing an undermining effect of rewards

Few studies have examined the impact of rewarding health behavior change with a specific focus on their potential to undermine intrinsic motivation.

In a field experiment, Charness and Gneezy (2009) incentivized college students for gym attendance and found students who had received contingent incentives attended the gym more frequently than the control group after incentives were removed. This result is opposite to the effect that would be predicted by motivation crowding theory. While acknowledging the limits of gym attendance as an outcome measure, the result in fact supports motivation “crowding in,” attributed by the authors to habit formation.

In a randomized controlled trial with children, Cooke et al. (2011) measured the effect of exposure and rewards (tangible stickers and intangible praise) on intake and liking of a vegetable up to 3 months after the intervention. Liking of the food can be seen as similar to Deci’s (1971) definition of intrinsic motivation. Children who had received tangible rewards consumed more of the vegetable than all other groups; they liked the vegetable equally well as other exposure groups, and more than the control group. This evidence does not support an undermining effect of the tangible incentive. Although motivation is a very unreliable predictor of behavior (Webb & Sheeran, 2006), tastes and preferences formed at this early age are likely to have repercussions throughout life.

Ledgerwood and Petry (2006) assessed the impact of incentives on motivation in the context of substance use. Motivation was assessed using questions based on the stages of change model. The group receiving incentives did not differ in motivation from the group receiving no incentives up to 3 months after the incentives had stopped, hence there was no evidence in this study either that the incentives reduced motivation.

#### Studies indirectly assessing a crowding out effect: Randomized controlled trials (RCTs) **of financial incentive effectiveness**

Cahill and Perera (2011) reviewed evidence from 19 studies (RCTs and controlled trials) assessing the impact of financial incentives on smoking cessation (not including pregnant women) and concluded that financial incentives resulted in higher quit rates while the incentive was in place. This is evidence in the opposite direction from that which would be predicted by motivation crowding theory. There was no difference between the incentivized and control group at follow-up, after the incentive was no longer in place, providing no evidence for an undermining effect of the incentive as operationalized in study designs building on cognitive evaluation theory. The largest study in the review (Volpp et al., 2009) stands out for finding higher quit rates in the previously incentivized group at 15-month follow-up, thus providing direct evidence against an undermining effect of the incentive.

A meta-analysis of nine trials on incentives for weight loss (Paul-Ebhohimhen & Avenell, 2008) found no difference between previously incentivized and control groups at follow-up, thus providing no evidence for reduced, or increased, motivation. In an RCT not included in this review (Volpp et al., 2008), the incentivized groups lost more weight than the control group during incentivization, and at 7-month follow-up participants in the previously incentivized groups, unlike those in the control group, weighed less than at baseline.

In a systematic review, Niza, Tung, and Marteau (2013, this issue) found no evidence that incentives for blood donations crowd out motivation while the incentive is in place; too few studies included a post-incentive follow-up to assess post-incentive impact. Randomized controlled trials about the effectiveness of incentives on adherence to medical treatment show that such incentives are sometimes effective, providing evidence against crowding out while the incentive is in place (e.g., Barnett, Sorensen, Wong, Haug, & Hall, 2009; Malotte, Hollingshead, & Larro, 2001; Tulsky et al., 2000). Long, Jahnle, Richardson, Loewenstein, and Volpp (2012) found no evidence for incentive effectiveness for blood glucose control in a 6-month RCT among African American veterans with diabetes, but outcomes in the incentive group were no worse than the control group (outcomes in the peer mentoring group, however, were better than in the control group). Barnett, Sorensen, Wong, Haug, and Hall (2009) also included a follow-up 4 weeks after removal of the incentive and found no difference between the previously incentivized and the control group, providing no evidence for a persisting undermining effect of the incentive.

Randomized controlled trials of incentives to attend screening for sexually transmitted diseases have found either no effect of the incentive (Low et al., 2007) or a positive effect (Malotte et al., 2004), hence generating no evidence to support the hypothesis that rewards reduce motivation. Kohler and Thornton (2012), however, report that in an incentive program in Malawi the randomly assigned incentive had no effect on HIV infections, but after it was paid out, risky sexual behavior increased by nine percentage points for men and decreased by seven percentage points for women. One possible mechanism suggested by the authors is that money may have enabled the men to purchase risky sex and enabled the women to avoid selling it, which would imply the incentive to have changed, for both men and women, the means to follow through on pre-existing motivation. In an RCT of incentives contingent on testing for sexually transmitted diseases in Tanzania, there was a positive effect of the incentive on self-reported behavior change (Packel, Dow, de Walque, Isdahl, & Majura, 2012), and a positive effect of incentives on self-reported motivation (de Walque, Dow, Medlin, & Nathan, 2012). Although these endpoints are not reliable indicators of behavior change, they provide evidence against crowding out.

### Comparison of Health-Related Behaviors With Behaviors for Which an Undermining Effect has Been Demonstrated

There seems to be very little evidence of an undermining effect of rewards on health-related behaviors. We now compare health behaviors and those studied in the psychological and economic literatures reviewed above for which an undermining effect is frequently found, in order to identify possible differences that might explain the different findings.

Table 2 summarizes our comparison of key characteristics of the behaviors used in these former two literatures with the characteristics of the different health-related behaviors for which incentive schemes have been used. The last column shows whether evidence in each domain supports the existence of an undermining effect. Although the evidence bases vary in size and the extent to which they have focused on revealing reduced behavior in the face of rewards, the pattern of findings support the interpretation of the key characteristics as moderators: undermining effects of incentives depend on a high baseline level for simple tasks, and on an interpersonal conflict of interest in more complex behaviors, neither of which are dominant for health-related behaviors that are targets for incentive schemes. For health-related behaviors that rely on self-control, incentives may enhance feelings of competence and might actually increase motivation and behavior even post-incentive, though little evidence for this long-term effect exists.[Table-anchor tbl2]

#### Rewards would be offered to individuals with low rather than high initial behavior levels

The most important difference between health behaviors and those studied in the cognitive evaluation theory literature concerns levels of the pre-reward behaviors and intrinsic motivation, an established moderator of the undermining effect of rewards (Deci et al., 1999). Rewards for health related behaviors aim to increase low baseline levels of a particular behavior that enhances health. For example, rewards would be offered to smokers if they quit, to overweight people if they lose weight (by consuming fewer or expending more calories), to those who rarely engage in physical activity if they increase their levels of physical activity. As noted above, meta-analyses find that for “dull” and “boring” tasks for which subjects show low pre-reward persistence, a reward has either no undermining effect or even a small beneficial effect (Deci et al., 1999). We note that the intrinsic motivation component of enjoyment may still be high for some health-related behaviors such as physical activity that are nevertheless rarely engaged in due to a number of internal or external barriers—the experience of enjoying running on the rare occasions that one does it. This is a result of the more complex set of motivations for health-related behaviors, for which high or low behavior levels do not directly map onto high or low intrinsic motivation, as is the case for the simple tasks without external benefits that are used in cognitive evaluation theory studies. However, in cognitive evaluation theory itself, operationalization of crowding out using behavioral measures takes precedence over measures of task enjoyment. The complex nature of motivation also means that health-related behaviors are practically never engaged in only for their own sake, but are at least partly motivated by considerations about outcomes such as improved fitness or attractiveness, so none of them meet the CET definition of high intrinsic motivation under any circumstances of which we can think.

#### Many health-related behaviors involve problems of self-control

Many health-related behaviors typically involve a conflict of considerations for long-term outcomes such as improved fitness with preferences for outcomes close in time, such as staying on the couch to watch television. An individual must trade these conflicting preferences off against each other when deciding what to do. It is well established that these trade-offs are temporally inconsistent (Frederick, Loewenstein, & O’Donoghue, 2002): When the sooner benefit is immediately available, it is preferred more than the individual would have predicted. Plans not to eat dessert are overthrown when it is on the table. The change in predicted behavior as the temptation becomes imminent is known as a problem of impulse control, or self-control (Ainslie, 1975; Baumeister, Heatherton, & Tice, 1994). As far as we are aware, no studies in the context of cognitive evaluation theory involve behaviors that challenge impulse control in this way. For health behaviors that rely on self-control, effective rewards might plausibly enhance perceived competence, making an undermining effect of the reward less likely, and a positive effect on behavior more likely. This effect is, for example, found in cognitive evaluation theory studies that involve verbal praise for difficult tasks (Deci et al., 1999). An effective incentive may work by adding to the delayed benefits of healthy behavior in a way that is just large enough, and just certain enough, and just close enough in time, to tilt behavior away from the option conferring immediate benefit, thereby effectively training self control mechanisms.

#### An interpersonal conflict of interest is less prominent for health-related behaviors

Evidence from economic studies about motivation crowding out relies mostly on behaviors and tasks for which narrow self-interest must be traded off against benefits to others. Before the reward is introduced, behavior is not maximally self-interested, demonstrating “intrinsic” motivation to contribute to others’ well-being. For example, employees work even when not supervised, and parents are not always late in picking up their children. When crowding out occurs, the behavior becomes more narrowly self-interested, that is, this intrinsic motivation is undermined; for example, parents are more often late. All three of the potential mechanisms suggested by Bowles (2008) for an undermining effect of incentives in addition to cognitive evaluation theory rely on this conflict of interest: framing of the decision as one where self-interest is the appropriate guiding principle; the incentive system shaping long-term narrowly self-interested preferences; the incentive conveying information, for example about lack of trust in employee work effort. Also based on a conflict of interest is the explanation offered by Heyman and Ariely (2004) whereby a situation requiring altruistic behavior that was previously situated in the “social market,” where no payment is expected, shifts to the “money market,” where narrow self-interest dictates behavior and payment for altruistic behavior is or is not high enough.

This structure of conflicting interests resembles the plausible motivation structure for some, but certainly not for all, health behaviors. It most closely resembles the case for blood and organ donation, where narrow self-interest is not at all served by the behavior. Behaviors such as disease screening or vaccinations for communicable diseases benefit both the individual and others (through herd immunity). These behaviors are most likely primarily motivated by interest in the health benefits to oneself, but may additionally be motivated by considerations for benefits to others. Rewards for donation, screening, and vaccination decisions might therefore be seen by those targeted as direct attempts by others (society, the health service, the organ recipient) to buy these benefits from them—a shift to the money market. Crowding out might occur. However, Frey and Jegen (2001) note that where crowding out occurs, the offer of an incentive reduces behavior compared with the situation in which no incentive is offered, but once an incentive is offered, increasing payment increases behavior. From a policy perspective, the motivating effect of a large enough incentive might be worth it, depending on how much beneficial behavior is gained.

For most behaviors that confer a health benefit when sustained in the long term, such as smoking cessation, weight loss, and physical activity, a conflict of interest between different parties is much less prominent. Harms to others from some unhealthy behaviors certainly exist. The most prominent example concerns the hazards of second-hand smoke, which has been used to motivate behavior change and legislation restricting where smoking can occur. In such cases, the offer of an incentive may be perceived by those targeted as a payment, for example from the state, to further the interest of others worthy of protection, or its own interests of reducing health care costs. However, for most of these behaviors, the prominent conflict of interest is less between different persons and more between different outcomes within the person him- or herself. Given that a large proportion of smokers and those who are overweight or inactive already have stated intentions to change these behaviors, the reward seems more likely to be seen as a welcome additional benefit for a behavior for which motivation already exists (Mantzari, Vogt, & Marteau, 2012). Although the intrapersonal, intertemporal conflict of interest can be conceptualized as one between present and future selves (Parfit, 1984), the reward would come from outside the person, a different situation from that of one party offering the reward in an interpersonal conflict of interest.

## Discussion

Evidence for the undermining effects of rewards in the psychological and economic literatures needs to be applied cautiously to health-related behaviors. The psychological literature on cognitive evaluation theory consistently reports an undermining effect of rewards only for behaviors for which initial intrinsic motivation, and initial behavior levels, are high. Such high pre-reward intrinsic motivation and behavior levels are not found in health-related behaviors that are the focus of incentive schemes. Studies in the economic literature that have found a crowding out effect usually involve a conflict of interest between different parties, with most mechanisms explaining this effect relying on this interpersonal conflict. Among the health-related behaviors, such a conflict of interest is evident for blood donation, for communicable disease screening, and possibly for smoking cessation, but it is less prominent for behaviors such as achieving weight loss or physical activity. Even where it is prominent, with one exception, we do not find evidence for rewards reducing motivation in randomized controlled trials on incentive effectiveness.

Whether rewards undermine the initial “intrinsic” (not narrowly self-interested) motivation for the targeted behavior may depend on whether the reward is seen as an attempt to “purchase” the behavior from the individual in a “money market.” Yet even where this is likely to be the case, such as for blood donation, evidence for lower levels of donation at a population level is not found (Niza, Tung, & Marteau, 2013, this issue). Because it is theoretically plausible that employer incentives for employee health behavior change (Volpp, Asch, Galvin, & Loewenstein, 2011) are perceived in this way more than incentives paid by researchers, employers may want to further minimize the risk of crowding out by framing the message of incentives as aiding individuals rather than as a bribe for better work performance. We note, however, that the trial showing the greatest long-term positive effect of incentives for smoking cessation (Volpp et al., 2009) was conducted in an employment setting and provides evidence against a crowding out effect. In all these cases, crowding out for some individuals may be outweighed by other individuals being sufficiently extrinsically motivated by the reward to perform the behavior when previously they did not. The question whether the reward should be offered could in this case be seen as a simple cost-effectiveness decision, informed by the impact of the incentive on the target behavior.

When rewards help tilt the balance of benefits in favor of the delayed, healthier outcome, they may shape behavior to bring it more in line with an underlying motivation that has previously been present albeit not manifest in behavior. If such a reward is perceived as confirming the individual’s autonomy rather than controlling his or her behavior, cognitive evaluation theory would predict it would enhance intrinsic motivation. Frey and Jegen (2001) likewise give a theoretical account for such “crowding in” to occur. Confirming this, the evidence on the effectiveness of financial incentive schemes targeting behaviors that involve problems of self-control does not reveal crowding out. Importantly, uncertainty remains about the design of financial incentive schemes, including the targeted behavior, that are most likely to lead to sustained behavior change, and for whom. Reviews and primary studies that address this question could usefully collect more evidence to substantiate or refute the analysis in this article regarding the limited number of contexts in which motivation crowding out is likely to occur when financial incentives are offered for health-related behavior change.

## Figures and Tables

**Table 1 tbl1:** Overview of Methods and Evidence From Cognitive Evaluation Theory and Motivation Crowding Theory, and Comparison With Health-Related Behaviors

	Intrinsic motivation definition	Operationalization and study design	Evidence base	Main findings	Health-related behaviors compared
Psychological literature: Cognitive evaluation theory	Doing the task exclusively for its own sake.	• Task and participants selected for high pre-reward engagement with task. • Between-subject design: one group receives reward, control group not. • Task persistence is compared between groups after the incentive is removed.	• Large evidence base. • Mostly lab experiments with children. • Simple tasks for which high task persistence plausibly reflects high intrinsic motivation.	• Tangible, expected rewards reduce behavior for interesting tasks (where behavior is initially high). • No reduced persistence in task for previously dull tasks (where little time is initially spent on task).	• More complex motivation. Pure intrinsic motivation unlikely. • Healthy behavior levels initially low (comparable with “boring” tasks). • Most behaviors subject to self-control problems.
Economic literature: Motivation crowding theory	Any of diverse motivations going in the opposite direction of narrow self-interest. Examples: altruism, “civic-mindedness”	• Behaviors are selected that are not maximally self-interested and benefit other parties. (e.g., volunteering, work effort). • Positive or negative incentive aims in the direction benefitting others. (e.g., rewarding volunteering; penalties for picking children up late). • Mostly between-subject, but also within-subject designs. • Motivation crowding out is said to occur when the incentive has an effect opposite of relative price effect, while the incentive is in place or afterwards.	• Growing evidence base. • Diverse studies (lab and field) and more diverse behaviors than CET literature.	• Incentives can have an unexpected effect in the “wrong” direction. • This is attributed to crowding out of “intrinsic motivation,” not specifically defined. • Circumstances under which incentives have this effect, opposite to the relative price effect are not well understood. • While offering any incentive can have a bad effect vs. no incentive, offering more money then usually operates in the expected direction.	• A conflict of interest is not usually prominent. • Most behaviors subject to self-control problems.

**Table 2 tbl2:** Comparison of Key Characteristics of Behaviors in Studies of: Cognitive Evaluation Theory Studies, Motivation Crowding Theory Studies, and Incentives Schemes Targeting Health-Related Behaviors

Literature	Specific behavior	High initial pre-reward behavior levels?	Conflict of interest between parties?	Self-control problem?	Evidence for crowding out?
**Cognitive evaluation theory**		**Yes**	No	No	Yes
**Motivation crowding theory**		No	**Yes**	No	Yes
**Health-related behaviors**					
	Smoking cessation	No	**Yes**	**Yes**	**No**
	Reduced calorie consumption	No	No	**Yes**	No
	Increased physical activity	No	No	**Yes**	No
	Vaccination and screening:				
	• noncommunicable disease	No	No	No	No
	• communicable disease	No	**Yes**	No	**No**
	Blood donation	No	**Yes**	No	**No**
*Note*. Boldface is a rough indicator for strength of the evidence (last column) and strength of arguments for the yes/no characterization (other columns).					

## References

[c1] Ainslie G. (1975). Specious reward: A behavioral theory of impulsiveness and impulse control. Psychological Bulletin, 82, 463–496 doi:10.1037/h0076860 1099599

[c2] Barnett P. G., Sorensen J. L., Wong W., Haug N. A., & Hall S. M. (2009). Effect of incentives for medication adherence on health care use and costs in methadone patients with HIV. Drug and Alcohol Dependence, 100, 115–121 doi:10.1016/j.drugalcdep.2008.09.017 19054631 PMC2715338

[c3] Baumeister R. F., Heatherton T. F., & Tice D. M. (1994). Losing control: How and why people fail at self-regulation. San Diego, CA: Academic Press

[c4] Bowles S. (2008). Policies designed for self-interested citizens may undermine “the moral sentiments”: Evidence from economic experiments. Science, 320, 1605–1609 doi:10.1126/science.1152110 18566278

[c5] Cahill K., & Perera R. (2011). Competitions and incentives for smoking cessation. Cochrane Database of Systematic Reviews. doi:10.1002/14651858.CD004307.pub4 18646105

[c6] Cameron J. (2001). Negative effects of reward on intrinsic motivation—A limited phenomenon: Comment on Deci, Koestner, and Ryan (2001). Review of Educational Research, 71, 29–42 doi:10.3102/00346543071001029

[c7] Cameron J., Banko K. M., & Pierce W. D. (2001). Pervasive negative effects of rewards on intrinsic motivation: The myth continues. The Behavior Analyst, 24(1), 1–44 22478353 10.1007/BF03392017 PMC2731358

[c8] Cameron J., & Pierce W. D. (1994). Reinforcement, reward, and intrinsic motivation: A meta-analysis. Review of Educational Research, 64, 363–423 doi:10.3102/00346543064003363

[c9] Charness G., & Gneezy U. (2009). Incentives to exercise. Econometrica, 77, 909–931 doi:10.3982/ECTA7416

[c10] Cooke L. J., Chambers L. C., Añez E. V., Croker H. A., Boniface D., Yeomans M. R., & Wardle J. (2011). Eating for pleasure or profit: The effect of incentives on children’s enjoyment of vegetables. Psychological Science, 22, 190–196 doi:10.1177/0956797610394662 21191095 PMC3272376

[c11] Deci E. L. (1971). Effects of externally mediated rewards on intrinsic motivation. Journal of Personality and Social Psychology, 18, 105–115 doi:10.1037/h0030644

[c12] Deci E. L., Koestner R., & Ryan R. M. (1999). A meta-analytic review of experiments examining the effects of extrinsic rewards on intrinsic motivation. Psychological Bulletin, 125, 627–668 doi:10.1037/0033-2909.125.6.627 10589297

[c13] Deci E. L., Koestner R., & Ryan R. M. (2001). Extrinsic rewards and intrinsic motivation in education: Reconsidered once again. Review of Educational Research, 71(1), 1–27 doi:10.3102/00346543071001001

[c14] Deci E. L., & Ryan R. M. (1985). Intrinsic motivation and self-determination in human behavior. New York, NY: Plenum Press

[c15] Deci E. L., Ryan R. M., & Koestner R. (2001). The pervasive negative effects of rewards on intrinsic motivation: Response to Cameron (2001). Review of Educational Research, 71, 43–51 doi:10.3102/00346543071001043

[c16] de Walque D., Dow W. H., Medlin C., & Nathan R. (2012). Stimulating demand for AIDS prevention: Lessons from the RESPECT trial. National Bureau of Economic Research Working Paper Series, 17865

[c17] Frederick S., Loewenstein G., & O’Donoghue T. (2002). Time discounting and time preference: A critical review. Journal of Economic Literature, 40, 351–401 doi:10.1257/002205102320161311

[c18] Frey B. S. (1997). On the relationship between intrinsic and extrinsic work motivation. International Journal of Industrial Organization, 15, 427–439 doi:10.1016/S0167-7187(96)01028-4

[c19] Frey B. S., & Goette L. (1999). Does pay motivate volunteers? Working Paper Series, (Working Paper No. 7). Retrieved from http://e-collection.library.ethz.ch/eserv/eth:25512/eth-25512–01.pdf

[c20] Frey B. S., & Jegen R. (2001). Motivation crowding theory. Journal of Economic Surveys, 15, 589–611 doi:10.1111/1467-6419.00150

[c21] Frey B. S., & Oberholzer-Gee F. (1997). The cost of price incentives: An empirical analysis of motivation crowding-out. American Economic Association, 87, 746–755

[c22] Gneezy U., Meier S., & Rey-Biel P. (2011). When and why incentives (don’t) work to modify behavior. The Journal of Economic Perspectives, 25, 191–210 doi:10.1257/jep.25.4.191

[c23] Gneezy U., & Rustichini A. (2000). A fine is a price. The Journal of Legal Studies, 29, 1–17 doi:10.1086/468061

[c24] Heyman J., & Ariely D. (2004). Effort for payment: A tale of two markets. Psychological Science, 15, 787–793 doi:10.1111/j.0956-7976.2004.00757.x 15482452

[c25] Johnston M., & Sniehotta F. (2010). Financial incentives to change patient behaviour. Journal of Health Services Research and Policy, 15, 131–132 doi:10.1258/jhsrp.2010.010048 20555040

[c26] Kohler H-P., & Thornton R. L. (2012). Conditional cash transfers and HIV/AIDS prevention: Unconditionally promising? The World Bank Economic Review, 26, 165–190 doi:10.1093/wber/lhr041 24319306 PMC3849819

[c27] Ledgerwood D. M., & Petry N. M. (2006). Does contingency management affect motivation to change substance use? Drug and Alcohol Dependence, 83, 65–72 doi:10.1016/j.drugalcdep.2005.10.012 16310974

[c28] Lepper M. R., Greene D., & Nisbett R. E. (1973). Undermining children’s intrinsic interest with extrinsic reward: A test of the “overjustification” hypothesis. Journal of Personality and Social Psychology, 28, 129–137 doi:10.1037/h0035519

[c29] Lepper M. R., Henderlong J., & Gingras I. (1999). Understanding the effects of extrinsic rewards on intrinsic motivation—Uses and abuses of meta-analysis: Comment on Deci, Koestner, and Ryan (1999). Psychological Bulletin, 125, 669–676 doi:10.1037/0033-2909.125.6.669 10589298

[c30] Long J. A., Jahnle E. C., Richardson D. M., Loewenstein G., & Volpp K. G. (2012). Peer mentoring and financial incentives to improve glucose control in African American veterans: A randomized trial. Annals of Internal Medicine, 156, 416–424 doi:10.7326/0003-4819-156-6-201203200-00004 22431674 PMC3475415

[c31] Low N., McCarthy A., Macleod J., Salisbury C., Campbell R., Roberts T. E., . . . Group C. S. S. P. (2007). Epidemiological, social, diagnostic and economic evaluation of population screening for genital chlamydial infection. Health Technology Assessment, 11, iii-iv, ix-xii, 1–165 10.3310/hta11080 17311735

[c32] Malotte C. K., Hollingshead J. R., & Larro M. (2001). Incentives vs. outreach workers for latent tuberculosis treatment in drug users. American Journal of Preventive Medicine, 20, 103–107 doi:10.1016/S0749-3797(00)00283-X 11165450

[c33] Malotte C. K., Ledsky R., Hogben M., Larro M., Middlestadt S., St. Lawrence J. S., . . . The GCAP Study Group (2004). Comparison of methods to increase repeat testing in persons treated for gonorrhea and/or chlamydia at public sexually transmitted disease clinics. Sexually Transmitted Diseases, 31, 637–642 doi:10.1097/01.olq.0000143083.38684.9d 15502669

[c34] Mantzari E., Vogt F., & Marteau T. M. (2012). The effectiveness of financial incentives for smoking cessation during pregnancy: Is it from being paid or from the extra aid? BMC Pregnancy Childbirth, 12, 24 doi:10.1186/1471-2393-12-24 22471787 PMC3338379

[c35] Niza C., Tung B., & Marteau T. M. (2013). Incentivizing blood donation: Systematic review and meta-analysis to test Titmuss’s hypotheses. Health Psychology. 10.1037/a0032740 PMC3920088 24001244

[c36] Packel L., Dow W., de Walque D., Isdahl Z., & Majura A. (2012). Sexual behavior change intentions and actions in the context of a randomized trial of a conditional cash transfer for HIV prevention in Tanzania. World Bank Policy Research Working Paper, No. 5997.

[c37] Parfit D. (1984). Reasons and persons. Oxford, UK: Oxford University Press

[c38] Parke H., Ashcroft R., Brown R., Marteau T. M., & Seale C. (2011). Financial incentives to encourage healthy behaviour: An analysis of UK media coverage. Health Expectations. Advanced online publication doi:10.1111/j.1369-7625.2011.00719.x PMC4180042 21771227

[c39] Paul-Ebhohimhen V., & Avenell A. (2008). Systematic review of the use of financial incentives in treatments for obesity and overweight. Obesity Reviews, 9, 355–367 doi:10.1111/j.1467-789X.2007.00409.x 17956546

[c40] Prendergast C. (1999). The provision of incentives in firms. Journal of Economic Literature, 37, 7–63 doi:10.1257/jel.37.1.7

[c41] Promberger M., Brown R. C. H., Ashcroft R. E., & Marteau T. M. (2011). Acceptability of financial incentives to improve health outcomes in U.K. and U.S. samples. Journal of Medical Ethics, 37, 682–687 doi:10.1136/jme.2010.039347 21670321 PMC3198007

[c42] Titmuss R. (1970). The gift relationship: From human blood to social policy. London, UK: George Allen and Unwin

[c43] Tulsky J., Pilote L., Hahn J. A., Zolopa A. J., Burke M., Chesney M., & Moss A. R. (2000). Adherence to isoniazid prophylaxis in the homeless: A randomized controlled trial. Archives of Internal Medicine, 160, 697–702 doi:10.1001/archinte.160.5.697 10724056

[c44] Volpp K. G., Asch D., Galvin R., & Loewenstein G. (2011). Redesigning employee health incentives—Lessons from behavioral economics. The New England Journal of Medicine, 365, 388–390 doi:10.1056/NEJMp1105966 21812669 PMC3696722

[c45] Volpp K. G., John L., Troxel A., Norton L., Fassbender J., & Loewenstein G. (2008). Financial incentive-based approaches for weight loss: A randomised trial. Journal of the American Medical Association, 300, 2631–2637 doi:10.1001/jama.2008.804 19066383 PMC3583583

[c46] Volpp K. G., Troxel A., Pauly M., Glick H., Puig A., Asch D., . . . Audrain-Mcgovern J. (2009). A randomized, controlled trial of financial incentives for smoking cessation. The New England Journal of Medicine, 360, 699–709 doi:10.1056/NEJMsa0806819 19213683

[c47] Webb T. L., & Sheeran P. (2006). Does changing behavioral intentions engender behavior change? A meta-analysis of the experimental evidence. Psychological Bulletin, 132, 249–268 16536643 10.1037/0033-2909.132.2.249

